# Validity and reliability of Turkish version of the Food–Mood Questionnaire for university students

**DOI:** 10.1017/S1368980021004377

**Published:** 2022-04

**Authors:** Nazlı Nur Aslan Çin, Betül Şeref, Ayşe Özfer Özçelik, Serdar Atav, Lina Begdache

**Affiliations:** 1 Ankara University, Faculty of Health Sciences, Department of Nutrition and Dietetics, Ankara, Turkey; 2 Karamanoglu Mehmetbey University, Faculty of Health Sciences, Department of Nutrition and Dietetics, Karaman, Turkey; 3 Binghamton University, Decker College of Nursing and Health Sciences, Binghamton, NY, USA; 4 Binghamton University, Department of Health and Wellness Studies, Binghamton, NY, USA

**Keywords:** Dietary pattern, Mental distress, Reliability, Validation

## Abstract

**Objective:**

This study aims to evaluate the validity and reliability of the Turkish version of the Food–Mood Questionnaire (FMQ) for university students.

**Design:**

The original questionnaire developed by Begdache *et al.* (2019) was modified and translated into Turkish. The content validation ratio (CVR) and the content validity index (CVI) were used for content validity assessment. The construct validity was assessed by exploratory factor analysis and confirmatory factor analysis (CFA) on data collected on university students who completed the survey online. Pearson’s correlation coefficients and Cronbach’s *α* were used to assess reliability and validity (*P* < 0·05).

**Setting:**

This study was conducted at five different universities in Turkey.

**Participants:**

A total of 251 (67 males and 184 females) undergraduate students participated in the study. Of these, seventy-five students completed a pre- and post-test assessment.

**Results:**

In the current study, 251 university students with a mean age of 21·9 ± 4·1 years participated. The mean CVR and CVI were 0·96 and 0·98, respectively. Factor loadings ranged from 0·341 to 0·863, and item total score correlations ranged from 0·142 to 0·749. Cronbach’s *α* coefficient was 0·633 for the whole scale. Five factors were extracted that had a good fit in CFA (*χ* ^2^/DF = 1·37, root mean error of approximation: 0·039, goodness-of-fit index: 0·911 and comparative fit index: 0·933).

**Conclusions:**

The Turkish FMQ is a valid and a reliable tool for university students. FMQ can be used by clinicians or researchers to examine the mental distress and dietary patterns of university students. Further testing of the FMQ is required for validation in the general population.

A decline in mental health, which directly impacts physical health and well-being, poses a risk in terms of non-communicable diseases, public health and health care costs^(1)^. For this reason, lifestyle changes, which are among the modifiable risk factors, could enhance mental well-being prophylactically as well as therapeutically^(2)^. In recent years, research on mental distress has reported associations with nutritional factors such as dietary patterns, dietary habits, dietary intake and food insecurity^(3–6)^. Although these associations may be complex, it is believed that nutrients impact mental health through direct mechanisms such as modulation of neurotransmitters and indirectly through epigenetic modifications as well as neurogenesis and neuroplasticity^(7,8)^. Therefore, malnutrition or an imbalanced diet is associated with the underlying pathophysiology of mental distress^(9)^. In fact, high-quality diet is implicated in the prevention and treatment of mental disorders^(10)^. The neurotransmitters that regulate human impulsions are closely related with the intake of several nutrients such as vitamins and essential amino acids^(11)^. Meta-analysis studies reported that marine-derived *n*-3 fatty acids regulate the neurotransmission of dopamine and serotonin, which aids in reducing depression^(12)^ and anxiety^(13)^. Thus, a poor diet leading to insufficient intake of nutrients leads to various degrees of mental distress^(10)^. Additionally, assessing the quality of the diet may support targeted therapeutic approaches to improve mental health^(14)^. Dietary habits have changed due to environmental influences such as urbanisation and globalisation. The Western-style diet, which includes high consumption of processed foods, saturated fat and sugar, has a negative impact on mental health as well as many metabolic diseases^(15,16)^. In fact, high scores in the Western-style diet model are associated with depressive symptoms^(17)^. A study of a Western-style diet determined a reduction in hippocampal-dependent learning and memory and a loss of appetite control after 3 weeks of being on the diet^(18)^.

On the other hand, research showed that regular adherence to a healthy diet supports hippocampal neurogenesis, which is linked to decreased risk of depression and enhanced mood^(19)^. Fruit and vegetable intake is the main way of achieving a healthy diet associated with greater mental well-being in young individuals^(20)^. In a systematic review, which included studies on the Mediterranean diet and mental health, individuals with higher dietary compliance had a lower risk of depression; and lower consumption of vegetables and fruits was associated with a higher perceived stress score^(21)^. Parletta *et al.* (2019) showed that the Mediterranean diet supplemented with fish oil positively affected the mental health of individuals with depression^(22)^. These studies suggest that a healthy diet has positive effects on decreasing mental distress and point to the need to evaluate dietary patterns and mental health.

University students may encounter mental distress such as anxiety and depressive disorders due to difficulties balancing social and academic life^(23)^. The WHO Mental Health Surveys reported that one-fifth (20·3 %) of college students exhibit a form of a mental disorder^(24)^. It is estimated that 12–46 % of college students face various degrees of mental disorders each year^(24–26)^. In Turkey, 11 % of university students suffer from various types of mental disorders^(27)^. Since diet is potentially the first line of defence against mental health ailments, it is necessary to assess college students’ dietary intake in relation to mental distress. However, such assessment tools are lacking in the Turkish language. Therefore, to fill a gap in the literature, the current study aims to adapt the Food–Mood Questionnaire (FMQ) developed by Begdache *et al.* ^(28)^ to the Turkish language and evaluate its validity and reliability in the Turkish college student population.

## Methods

### Study group

Data were collected between January 2021 and April 2021 from 251 undergraduate students from 5 different Turkish universities and colleges through an online questionnaire. The questionnaire was sent to various social and professional groups via social media. Participants in the study were required to be 18 years or older, enrolled as a student at a university and using the Turkish language for communication. In this study, we did not create a homogeneous group, as we wanted to evaluate the compliance of all university students to the scale. Thus, as in the original Begdache study, data were collected regardless of the age and division of the students.

### Turkish adaptation protocol

A permission to translate FMQ was secured via an email communication with Begdache *et al.* The English version of the FMQ was translated to Turkish. The translation was done the forward–backward translation method. With the forward translation, two translators with fluent English translated the questionnaire into Turkish, unaware of each other. The two versions were checked and any discrepancies were solved collaboratively by the research team. It was translated back into English by another bilingual speaker who did not know the English version. The final translated version of the questionnaire was forward to a representative of Begdache *et al.* to confirm its content accuracy.

### Assessment of content validity

A group of eight nutritionists, a psychiatrist and a psychologist tested the content validity of the FMQ. The experts were asked to evaluate the simplicity, clarity, relevance and necessity of each question. The content validation ratio (CVR) was calculated for each item with the Lawshe method^(29)^. CVR is an item statistic based on content validity that evaluates whether the items are suitable for the scale. The number of experts who gave the appropriate answer to the item is divided by the total number of experts and it is calculated by subtracting one from this value. If all of the participants rate any item in the scale as ‘Suitable’, the CVR value of that item becomes one^(30)^. The content validity index (CVI) is calculated for the whole test after the items are defined including the scale with the determination of the CVR. In this case, the CVI value is obtained by calculating the average of the CVR values of the items decided to be included in the scale^(31)^. The lowest acceptable CVR value for ten experts was 0·62^(30)^. The CVI was also computed for each item and the lowest acceptable agreement point was 0·76 in this study^(31)^.

### Assessment of construct validity

**Fig. 1:**
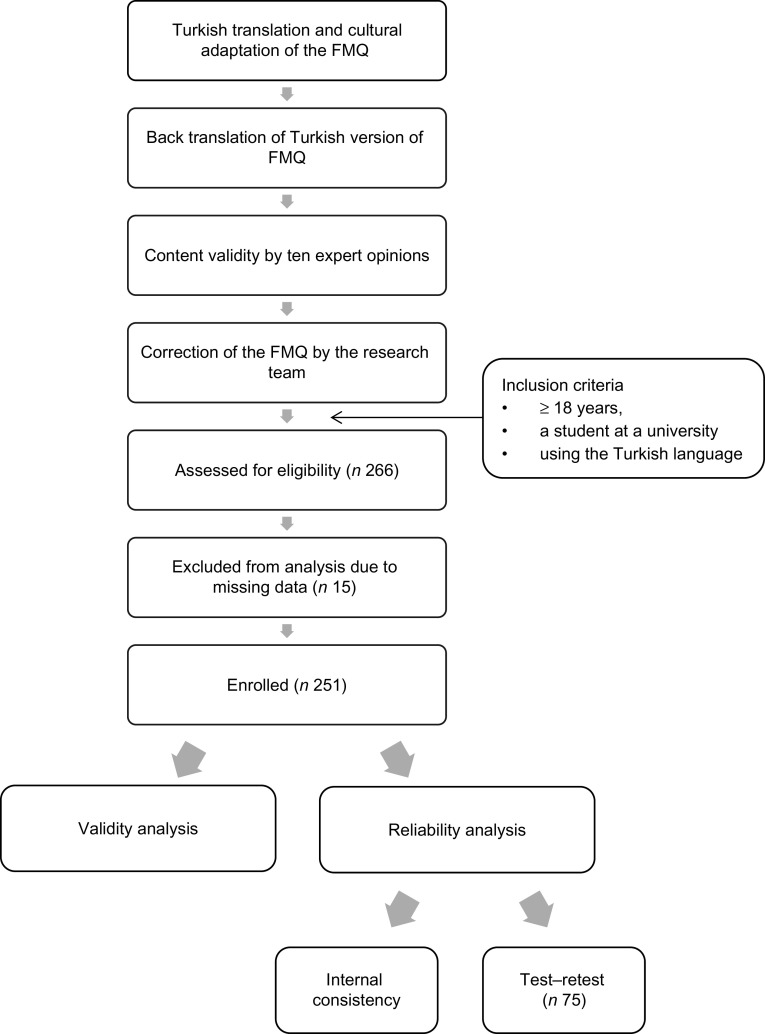
Flow diagram of the conduct of the study. FMQ, Food–Mood Questionnaire

For this group of university students, construct validity of the scale was assessed using factor analysis. The sample size was calculated using the Gorsuch rule with the N:P ratio of 5, where ‘N’ is the minimum sample size and ‘P’ is the number of questions^(32)^. In the current study, ‘P’ was 22. Therefore, a least 110 participants were necessary in order to achieve an adequate sample size. A total of 251 university student responses were collected due to students with missing information in their data (*n* 15) (Fig. 1). The data collected included age, sex, academic division, smoking status and alcohol consumption.

The students were requested to complete the translated version of the FMQ. The original questionnaire has twenty-one questions on the six-point Likert scale (ranging from 0 = none of the time to 6 = more than four times). The original questionnaire consisted of five sub-dimensions. These sub-dimensions were mental distress, breakfast pattern, healthy pattern, Western-diet pattern and supplement pattern. The scale is additive; therefore, the total score is typically used. However, the Turkish questionnaire consists of twenty-two questions due to cultural eating differences. After a thorough analysis of the Turkish diet, it was suggested to add this item (sugary foods such as sweets, candy or chocolate) to better capture the impact of low-nutrient-dense food that may affect neurotransmission. The rationale behind the addition is that food high in simple carbohydrates causes fluctuation in blood sugar, which disrupts optimal levels of dopamine and serotonin, the two key neurotransmitters that contribute to the regulation of mood and mental health^(33)^. Since this food is highly consumed in this part of the world, this question is included the scale, with the permission of the original FMQ developer. Exploratory factor analysis was performed by principal component analysis with varimax rotation to test the factor structures of the twenty-two questions, following the methods used in the original Begdache study. The Kaiser–Meyer–Olkin (KMO) test and Bartlett’s test of sphericity were utilised for testing the sample adequacy. Items with factor loads below 0·30 or overlapping were excluded from the scale. For the internal consistency, item analysis and reliability coefficient (Cronbach’s *α*) were performed.

### Data analysis

Confirmatory factor analysis (CFA) was performed with AMOS version 21. SPSS software version 25.0. was used for all statistical analyses. Model fit was evaluated using *χ* ^2^, root mean error of approximation (RMSEA), goodness-of-fit index (GFI) and comparative fit index (CFI). Chi-square *P* value > 0·05, RMSEA < 0·08, GFI and CFI > 0·9 are acceptable values^(34,35)^. The factorial structure of the FMQ was examined by exploratory factor analysis. The standardised parameter Cronbach’s *α* was used for the internal consistency of the scale. The test–retest reliability of the scale was re-evaluated 4 weeks later.

## Results

In this study, 251 university students (67 males and 184 females) participated in the construct validity FMQ. Participants were students in the health sciences and other disciplines such as engineering and business administration from five different universities in Turkey. The baseline characteristics of the participants are provided in Table 1. The mean age of students was 21·9 ± 4·1 years and 73·3 % of them were females.

**Table 1:** Baseline characteristics of university students

Demographic	*n*	%
Age (years)		
Mean	21·9	
sd	4·1	
< 30 years	231	92·0 %
≥30 years	20	8·0 %
Sex		
Female	184	73·3 %
Male	67	26·7 %
Smoking status	33	13·1 %
Alcohol consumption	55	21·9 %
Division		
Health sciences	215	85·7
Other	36	14·3

The face and content validity scores of the questionnaire are shown in Table 2. The mean CVR and CVI of the FMQ were 0·96 and 0·98, respectively.

**Table 2:** The results for the content validity of the Turkish version of the FMQ

Items	CVR*	CVI†
Q17 = Depressed	0·80	0·90
Q18 = Hopeless	1	1
Q19 = Worthless	1	1
Q20 = Nervous	1	1
Q21 = Effort	1	1
Q22 = Restless or fidgety	1	1
Q11 = Beans	1	1
Q10 = Vegetables	1	1
Q3 = Whole grain	1	1
Q12 = Fish	1	1
Q1 = Exercise	1	1
Q4 = Dairy	1	1
Q6 = Fruits	0·80	0·90
Q7 = Nuts	1	1
Q2 = Breakfast	1	1
Q5 = Caffeine	0·80	0·90
Q9 = Meat/chicken/turkey	1	1
Q8 = Rice/pasta	1	1
Q13 = Fast foods	0·80	0·90
Q14 = Candy	1	1
Q15 = Multivitamin supplements	1	1
Q16 = Fish oil supplements	1	1
Total	0·96	0·98

FMQ, FMQ, Food–Mood Questionnaire; CVR, content validity ratio; CVI, content validity index.

* Values > 0·62 are acceptable.

† Values > 0·79 are acceptable.

The results of the factor analysis are shown in Table 3. The factor analysis was conducted using principal components extraction with varimax rotation. The suitability of the analysis was confirmed by identified indicators of the high degree of interrelationship between the variables: Bartlett’s test of sphericity was *χ* ^2^ = 1308; *P* = 0·00, and the KMO index was 0·78. In this study, the twenty-two items in the Turkish version of the FMQ produced five factors similar to the original questionnaire. The factor load, which shows the relationship of each item with the total score, was over 0·30, and five factors accounted for 50·4 % of the variance. The extracted factors were kept as defined in the original paper: mental distress; healthy pattern; breakfast pattern; Western-diet pattern and supplement pattern.

**Table 3:** Results of explanatory factor analysis of the Turkish version of the FMQ

Items	Factor 1: mental distress	Factor 2: healthy pattern	Factor 3: breakfast pattern	Factor 4: Western pattern	Factor 5: supplement pattern
Q17 = Depressed	0·843				
Q18 = Hopeless	0·863				
Q19 = Worthless	0·771				
Q20 = Nervous	0·811				
Q21 = Effort	0·594				
Q22 = Restless or fidgety	0·810				
Q11 = Beans		0·642			
Q10 = Vegetables		0·743			
Q3 = Whole grain		0·601			
Q12 = Fish		0·341			
Q1 = Exercise		0·560			
Q4 = Dairy			0·679		
Q6 = Fruits		0·405	0·429		
Q7 = Nuts		0·368	0·474		
Q2 = Breakfast			0·628		
Q5 = Caffeine				0·304	
Q9 = Meat/chicken/turkey				0·498	
Q8 = Rice/pasta				0·609	
Q13 = Fast foods				0·623	
Q14 = Candy				0·674	
Q15 = Multivitamin supplements					0·814
Q16 = Fish oil supplements					0·776
Eigenvalue	3·95	2·64	1·40	1·83	1·27
The percentage of variance explanation	17·4	10·2	8·7	7·5	6·6

FMQ, Food–Mood Questionnaire.

Extraction method: principal component analysis. Rotation method: varimax with Kaiser normalization.

The questions are given according to their factor loads.

Kaiser–Meyer–Olkin Index = 0·78; *χ* ^2^ = 1308; Bartlett’s test of sphericity *P* < 0·001.

**Fig. 2:**
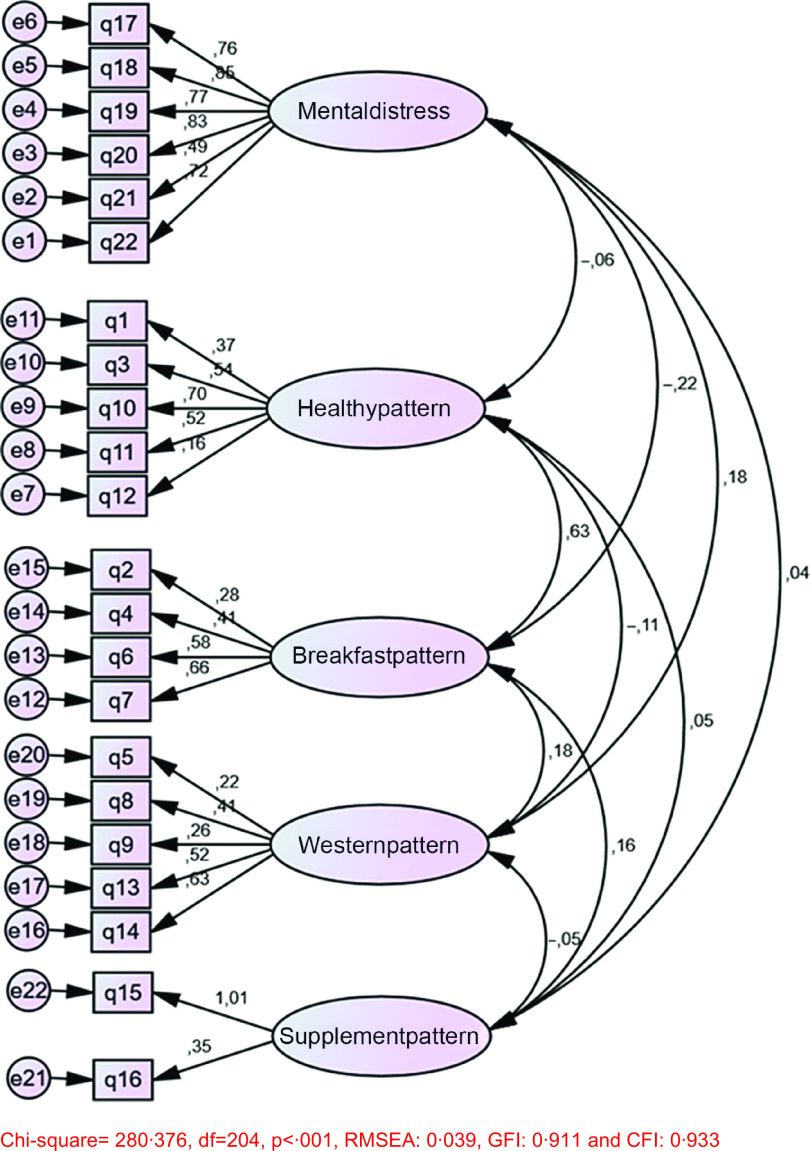
Results of confirmatory analyses of the Turkish version of the FMQ. FMQ, Food–Mood Questionnaire

The results of the CFA and goodness-of-fit indicators are shown in Fig. 2 and Table 4, respectively. According to these results, the five-factor model had a good fit in the Turkish data (*χ* ^2^/DF = 1·37, RMSEA: 0·039, GFI: 0·911 and CFI: 0·933).

**Table 4:** Results of confirmatory factor analysis of the Turkish version of the FMQ

Fit statistic	Value	Criteria
*χ* ^2^/DF	1·37	<5
Root mean squared error of approximation (RMSEA)	0·039	<0·08
Goodness-of-fit index (GFI)	0·911	≥0·90
Comparative fit index (CFI)	0·933	≥0·90

FMQ, Food–Mood Questionnaire.

*χ* ^2^ = 280·376, df = 204, *P* < 0·001.

The internal consistency level among the items of the FMQ, item-total correlations and Cronbach’s *α* internal consistency coefficients are presented in Table 5. The internal consistency coefficient using the Cronbach’s *α* was 0·633, supporting the scale reliability. Cronbach’s *α* coefficients are between 0 and 1. A coefficient close to 1 means that the scale is perfectly reliable. As described in Table 5, the corrected-item-total score correlation (correlation of all items with the total score) is positive and above 0·40. The corrected-item-total correlation is performed to show the consistency between one item and other items in a scale. It means that the scale can measure the food–mood relationship comprehensively. Pearson’s correlation coefficients were calculated on the scores of the seventy-five participants who completed the pre- and post-tests. The correlation coefficients are positive and above 0·40. Therefore, none of the items should be removed from the scale as evidenced by the Cronbach *α*’s under ‘the Cronbach’s *α* if item deleted’ column (Table 5).

**Table 5:** Results of the internal consistency analysis of the Turkish version of the FMQ

Items	Corrected-item-total score correlation (*n* 251)	Correlations coefficients *r* (*n* 75)	Cronbach’s *α* if item deleted (*n* 251)
Mental distress			
Depressed	0·749	0·580*	0·838
Hopeless	0·771	0·611*	0·834
Worthless	0·673	0·517*	0·852
Nervous	0·714	0·552*	0·847
Effort	0·469	0·535*	0·887
Restless or fidgety	0·673	0·569*	0·852
Healthy pattern			
Beans	0·376	0·633*	0·498
Vegetables	0·457	0·737*	0·427
Whole grain	0·381	0·578*	0·479
Fish	0·142	0·723*	0·588
Exercise	0·318	0·552*	0·535
Breakfast pattern			
Dairy	0·325	0·566*	0·497
Fruits	0·363	0·578*	0·456
Nuts	0·429	0·761*	0·390
Breakfast	0·252	0·699*	0·547
Western pattern			
Caffeine	0·168	0·410*	0·511
Meat/chicken/turkey	0·221	0·735*	0·468
Rice/pasta	0·322	0·579*	0·409
Fast foods	0·290	0·543*	0·426
Candy	0·362	0·576*	0·368
Supplement pattern			
Multivitamin supplements	0·355	0·539*	–
Fish oil supplements	0·355	0·912*	–

FMQ, Food–Mood Questionnaire.

* *P* < 0·001 (two-tailed).

## Discussion

The FMQ is a short, valid and reliable scale developed to evaluate the impact of food on mental distress^(28)^. A comprehensive, valid and reliable measuring tool in the Turkish language that evaluates dietary intake associated with mental distress is lacking. Therefore, the validity and reliability analysis of this FMQ in the Turkish language fills a gap in the literature.

Ten experts evaluated the content validity of the scale, and CVR and CVI values were used to assess the expert opinions. CVR and CVI values above 0·80 indicate agreement among expert opinions^(32)^. Accordingly, the CVR and CVI results determined that there was agreement among the experts in this study. The scale efficiently measured the topic, and content validity was achieved. The Bartlett sphericity test and KMO value were performed to determine that the data were appropriate and sufficient for factor analysis. The Bartlett’s sphericity test value should be *P* < 0·05 (statistically significant) and the KMO value should be at least 0·60^(34)^. In this study, Bartlett’s sphericity test value was statistically significant (*P* < 0·05), and the KMO value (0·78) was >0·60. Therefore, we conclude that the data and the sample size were adequate for the factor analysis.

According to the factor analysis model, five factors were removed that confirm the structure of the FMQ. Likewise, the original questionnaire consisted of five factors. The first factor, mental distress, comprises six items; the second one, a healthy pattern, comprises five items; the third one, breakfast pattern, comprises four items; the fourth one, Western-diet pattern, comprises five items; and the last factor, supplement pattern, comprises two items. The Turkish questionnaire provides the construct validity of the original questionnaire. According to the combination of items, mental distress and supplement pattern factors were found to be the same in the Turkish version as in the original English version. However, in the Turkish version, questions on fruits and nuts were loaded on factor 3 (healthy pattern) instead of factor 2 (breakfast pattern) in the original version; the question of the whole grain loaded in factor 2 (healthy pattern) instead of factor 3 (breakfast pattern) in the original version; and the question of caffeine loaded in factor 4 (Western-diet pattern) instead of factor 3 (breakfast pattern) in the original version. These inconsistencies between the results of studies stem from the food cultural differences of the studied populations. In Turkish culture, fruit and nuts are not consumed only at breakfast but throughout the day as part of main dishes (as garnish) or as snacks between meals. Similarly, in Turkish culture, whole-grain products are part of meals and not limited to breakfast items.

For the validity and reliability of the scale, literature suggested that the structure determined by exploratory factor analysis should also be examined with CFA^(34,35)^. CFA for the present study indicated that the fit indexes (GFI and CFI) were >0·90, the RMSEA was <0·08 and the *χ* ^2^ value/df was <5. Along with these results, the relationship between the scale and sub-dimensions was highly significant. Current literature suggests that a good fit indicator is defined as a division of >0·85, *χ* ^2^/df of the model fit indicators is <5 and RMSEA < 0·08^(34–36)^. The CFA results obtained were consistent with the previous studies. Begdache *et al.* ^(28)^ had not performed a CFA analysis with the original scale, hence that was not possible to compare with current findings. The CFA results confirmed that the data set was compatible with the model and the five-factor structure, the sub-dimensions were connected to the scale, and the items in each sub-scale adequately defined their own factor. The exploratory factor analysis and CFA results of this study demonstrated the validity of the scale.

The Cronbach’s *α* coefficient is another method in reliability analysis^(34)^. Cronbach’s *α* values >0·60 specify that the scale is reliable^(35)^. In addition, if it is greater than 0·70, the test is highly reliable^(37)^. The reliability coefficient of the whole scale in the study is 0·633. A Cronbach’s *α* greater than 0·60 was considered reliable both in this study and in other studies^(38,39)^. Begdache *et al.* reported that the total Cronbach’s *α* values of the scale were >0·70. The Cronbach’s *α* value of 0·633 in this study shows similar features to the original scale^(28)^. The scale shows similar structure to the original version and exhibits a good internal consistency. Item analysis is another method used to determine internal consistency^(40)^. In this study, the correlation coefficients between sub-dimensions and the total score were between 0·14 and 0·74. Following the recommendations from literature, items with weak correlations were not removed as their removal did not affect the Cronbach’s *α* ^(34–36)^. The test–retest method is another reliability criterion^(41)^. In this study, both the pre- and post-correlation coefficients of the scale items vary between 0·41 and 0·76, supporting the test–retest reliability of the scale.

The current study introduces a new tool in the Turkish language to the current literature on mental distress and dietary patterns. It may guide future research in mental distress and dietary patterns for the Turkish population. Although this paper provided significant data on the relationship between mental distress and dietary patterns, there were a few limitations. Although the use of an online survey for data collection was effective, there might have been risk for a response bias as a convenience sample was used. The majority of the participants were female students which may cause a gender bias. However, this issue was controlled as a confounding factor in the analysis. In addition, the fact that the study population consisted of only university students would affect generalisability to the general adult population.

## Conclusion

The results of our analyses evaluating the food and mood behaviours of university students in Turkey showed that the FMQ is a valid and a reliable tool for use in this specific population. The Turkish FMQ version can be useful in counselling services to university students who experience an increase in anxiety and depressive disorders. It may potentially increase compliance of use since it is a short and concise tool. It may support identification of mental distress cases among Turkish college students as well. The authors recommend further studies to determine the generalisability of the scale beyond the college population. In addition, future studies should examine the relationship between mental distress and diet of students from different neighbouring cultures using this scale.
